# “Morbus Mediterraneus" and its impact on medical care in Germany: the intersection of pain and racism

**DOI:** 10.1186/s12910-025-01321-2

**Published:** 2025-11-01

**Authors:** Sergio R. Pérez Rosal, Sonya C. Faber, Monnica T. Williams

**Affiliations:** 1 Universitätsklinikum Ruppin-Brandenburg, Neuruppin, Germany; 2 Medizinische Hochschule Brandenburg Theodor Fontane, Fehrbelliner Str. 38, Neuruppin, 16816 Germany; 3 School of Psychology, University of Ottawa, Ottawa, Canada

**Keywords:** Pain, Racism, Germany, Healthcare, Migration background, Bias

## Abstract

**Background and objective:**

In German healthcare, the colloquial term “Morbus Mediterraneus” is often used to dismiss pain complaints from racialized patients—particularly women of Mediterranean or non-White backgrounds—as exaggerated or dramatized. Although this label has no scientific basis, it perpetuates biased assumptions that lead to serious consequences, including undertreatment, misdiagnoses, and loss of trust in medical institutions. This paper provides a conceptual and literature-based analysis of how “Morbus Mediterraneus” reflects broader racist beliefs about pain tolerance, rooted in both colonial history and cultural norms in Germany.

**Methodology:**

This is a conceptual study drawing on previously published qualitative findings, historical records, and contemporary literature regarding racism, pain perception, and healthcare disparities. We review how confusion around racial terminology in German discourse impedes recognition and measurement of systemic racism. We integrate scholarship from critical race theory, intersectionality, and structural competence to highlight the deep-seated impact of racial biases on clinical decision-making.

**Results:**

Our analysis shows that “Morbus Mediterraneus” arises from a longstanding pattern of racialized medicine, where factors such as colonial research abuses, cultural misinterpretations of pain expression, and implicit provider biases converge. These biases systematically devalue the pain of racialized patients, especially women, and undermine patient–provider trust. Furthermore, we identify how gendered and racial stereotypes about emotional display and stoicism compound to create unique barriers to proper pain management.

**Conclusions:**

Confronting biases tied to “Morbus Mediterraneus” requires integrated reforms across medical education, clinical practice, and policy. We recommend mandatory anti-racism and structural competence training, greater racial diversity among healthcare workers, standardized pain-assessment protocols, and improved data collection on race and ethnicity. By acknowledging colonial legacies and cultural norms that shape pain perception, German healthcare can better address systemic racism, ensure equitable pain management, and ultimately improve patient outcomes for all.

## Introduction

In December 2020 German Representative Mirrianne Mahn recounted a troubling exchange with a physician while hospitalized for acute pain*.* “*I asked the doctor if the pain was normal, and he said pain is never normal, but you Black people can put up with it better.*” [53]. This anecdote, (fully described in Table 2) which she recorded from her hospital bed, illustrates the type of concerning treatment that racialized patients may encounter in German medical settings. Critically, it also highlights how racial stereotypes, particularly around pain tolerance, which have no biological basis, continue to influence clinical judgment and interpersonal care.

In some German medical settings, the term "*Morbus Mediterraneus*" is colloquially used as a mock diagnosis to label racialized patients, often women, from Mediterranean countries or with a migration background, but also racialized native Germans, who are perceived to exaggerate their pain or symptoms. The term combines the Latin word "Morbus" (disease) with "Mediterraneus" (Mediterranean), implicitly attributing a cultural or ethnic basis to the patients' expressions of pain.

This derogatory label reflects underlying racist and sexist prejudices, suggesting that certain racialized individuals are inherently more "hypersensitive" or emotionally demonstrative, or less stoic than their White German counterparts. The use of such terms not only undermines the patient-provider relationship but also has significant implications for the quality of medical care received by these patients.

This paper aims to critically examine the use of the term “Morbus Mediterraneus” as a manifestation of structural racism in German healthcare. It asks: How do racist biases shape pain management practices in Germany, and what historical, cultural, and institutional factors contribute to the ongoing use of racially coded language in medical care? To answer this question, the article draws on published qualitative research, historical examples, and literature on racism and pain to explore how racialized patients in Germany experience diagnostic dismissal and inadequate treatment.

### Significance of the issue

As part of a joint project on racism in healthcare funded by the German Federal Ministry of Education and Research, qualitative interviews and group discussions with patients and their families about their experiences in hospitals and rehabilitation facilities were conducted and analyzed. It was revealed that “the most significant experiences of racism in hospitals are attributions of dramatization and exaggeration of pain, embedded in a general perception of not being taken seriously and/or being ignored” [13]. The authors concluded that “racism in German inpatient healthcare represents a considerable psychosocial burden for patients and impairs the quality of treatment they receive.”

The perpetuation of stereotypes such as "Morbus Mediterraneus" in healthcare settings contributes to these systemic biases that adversely affect patient care. Patients who are not taken seriously may receive inadequate pain management, misdiagnoses, or delayed treatment, leading to worsening of their conditions and even life-threatening consequences. This issue is particularly pertinent in Germany, where a significant portion of the population comprises immigrants and their descendants, especially from Turkey, Italy, and other Balkan and Mediterranean countries. This issue is not confined to Germany; similar patterns of bias have been observed in Sweden, where patients' complaints are systemically devalued leading to suboptimal care [34].

Understanding the interplay between pain perception, cultural expression, and racism is crucial, for developing effective healthcare practices that are equitable and sensitive to the needs of everyone in Germany, especially given that perceived differences in pain expression are culturally and structurally shaped, not biologically determined.

### Structure of the article

This paper proceeds in six sections. First, it defines and contextualizes the term "Morbus Mediterraneus" within German medical discourse. Second, it explores the historical and colonial roots of racist beliefs in pain tolerance. Third, it examines contemporary clinical implications and disparities in pain management. Fourth, it analyzes cultural differences in pain expression, showing how misinterpretations reinforce racial stereotypes in medical contexts. Fifth, it discusses the psychological and intersectional consequences of racialized pain treatment, including the role of implicit bias in healthcare. Finally, it offers recommendations for medical education, clinical practice and policy to dismantle these embedded inequities.

### Positionality

When conducting research focused on race, ethnicity and culture, it is important to recognize the role of researcher positionality in the design, execution, and interpretation of any study as a means of mitigating bias**.** It also allows readers to appreciate the range of perspectives offered in the analysis of the topic under study [2].

The first author is a Guatemalan-German medical board-certified anesthesiologist and emergency doctor, holding a master's degree in neuroscience and psychology in training for cognitive-behavioral psychotherapy. The second author is an African American living in Germany and an experienced neuroscientist and pharmaceutical professional, specializing in clinical development and social justice. The last author is a national research chair, a registered clinical psychologist, and a Black American woman living in Canada. She has published over 200 peer-reviewed articles and 5 books, with a focus on mental health disparities and psychopathology, including prominent articles about racial bias. Their distinct cultural and professional backgrounds help shape the research questions and the interpretation of findings on systemic inequities in mental health.

## Understanding "Morbus Mediterraneus"

### Origin and meaning

"Morbus Mediterraneus" is not an actual medical diagnosis, but a colloquial term used by some healthcare professionals in Germany to label patients, typically from the Mediterranean region but also potentially any people of color, who are perceived to complain excessively about pain or symptoms. Though more commonly used with women, it can describe someone of any gender. Similar terms include "Morbus Bosporus" and "Mamma-mia-Syndrome," all of which ultimately serve to stereotype and minimize the health concerns of individuals based on their presumed ethnic background. This labeling reflects systemic racism in clinical settings.

To understand how such terminology gains traction, it is essential to explore how the concepts of race, ethnicity, and migration history are used, often inconsistently, in German discourse. These terms are frequently conflated, which contributes to misrecognition and the dismissal of racialized patients.

### Race is not the same as ethnicity

Race, ethnicity, and migration history are related but distinct concepts. Race refers to socially constructed categories based on physical traits such as skin color which governments and institutions have historically used to enforce frameworks of power and access. These definitions then in turn shape people's lived realities in ways that contribute to social hierarchies. It is not a biological or genetic distinction. There is no agency in its definition, that is individuals cannot decide for themselves to which race they belong. Ethnicity in contrast to race, is about shared cultural traits, language, traditions, or ancestry and may cross racial lines [1, 40]. It is malleable and self-defined, notably, two people of different races may share the same ethnicity. Ethnicities may or may not include shared genetic ancestry.

Racialized people refers to anyone living in a White supremacist (Western) society who is not White, i.e. people of color. BIPOC refers to Black, Indigenous, and other people of color (e.g., Asian, Latin American, and Arab people), who actually constitute the majority worldwide. As such, non-White people can also be referred to as people of the global majority (PGM). Referring to BIPOC as “minorities” is inaccurate and reinforces racial hierarchies, as these groups have been purposefully minoritized, despite being the global majority.

Germany has its own terms for diverse people groups according to its history and needs, recording 30% of its population as having a “migration background” (see Table 1 for definition) [61]. Migration history, as with all terms defining race, has been defined by the German Government and refers to a person’s or their family’s movement across borders, often focusing on the reasons (e.g., economic, political) and timing of migration. In practice, however, it functions less as a neutral demographic descriptor and more as a racialized category. Technically, it refers to non-White and people with at least one non-German parent, and includes those of Turkish heritage (Table 1). As such it is typically used as a euphemism for people of color, allowing racial distinctions to be made without naming race directly. This obscures the role of racism in social inequalities while reinforcing the perception that non-White people are perpetual outsiders.

**Table 1:** Terminology

Race	A socially constructed system based on perceived physical features (skin color/facial features), and presumed ancestry. Defined by governments and institutions not individuals. Used historically and currently to enforce hierarchies of power and access
Ethnicity	A self-identified affiliation based on shared culture, language, traditions, customs, religious practices, and/or historical experience. Ethnicities can cross racial lines and people of the same race can belong to different ethnic groups
Migration Background/History	Term used in Germany to describe people or families with at least one parent or grandparent born outside the country Often functions as a euphemism for race especially when applied selectively to non-White people while excluding White migrants
Structural racism	A system in which policies, institutional practices, and cultural representations perpetuate racial group inequity, often without overt individual intent
Internalized racism	When members of racialized groups accept and reproduce negative beliefs or stereotypes about their own group, often unconsciously
Aversive racism	A form of racism where individuals endorse equality explicitly but maintain implicit negative feelings or assumptions toward racialized groups. These biases typically surface in subtle ways, especially when behavior can be justified on non-racial grounds or goes unnoticed, allowing the individual to maintain a positive self-image while still engaging in biased actions
Dominative racism	Overt and intentional racism, such as racial slurs or violence, based on perceived superiority of one group over another
Symbolic- modern racism	Indirect forms of racial prejudice expressed through beliefs in moral inferiority or “cultural deficiency” (e.g., blaming racialized groups for structural inequality)
Micro- aggressions	Everyday verbal, behavioral, or environmental indignities — often unintentional — that communicate hostility or insult toward members of marginalized groups
Whiteness	a socio-political construct not an ethnicity, that refers to the social advantages and cultural norms associated with being perceived as White. Whiteness is reinforced through institutions, norms, and policies in Western societies
BIPOC	Acronym for Black, Indigenous, and people of color Emphasizes solidarity while recognizing that different racialized groups experience oppression in distinct ways
People of the Global Majority	A term used to describe people who are non-White globally, highlighting that they represent the majority of the world’s population and challenging deficit-based terms like “minority.”
These terms reflect commonly accepted definitions in public health and social science. Usage may vary across contexts and disciplines. Definitions informed by Gerhards et al. [29], Haeny et al. [33], and Comas-Díaz & Jacobsen [17]	

In Germany, the term “migration history” or “migration background” furthermore is selectively applied, excluding individuals who “migrate” from predominantly White countries, effectively making it a racial category rather than one based purely on migration. The reluctance to use the words "race" or “White” reflects an unwillingness to confront the social reality that, if a person’s skin is dark, they may never truly be considered German, even if their ancestors lived in Germany since the Renaissance, while those who are White are considered German regardless of their “migration history.” This is why “migration background,” reinforces racial distinctions. It creates a hierarchy of belonging, where Whiteness is equated with “Germanness,” while others are seen as “perpetual foreigners.”

This semantic confusion blurs distinctions between race and ethnicity, and obscures the effects of structural racism by masking it as cultural difference. This vocabulary gap not only hinders accurate assessment of disparities but also creates an illusion of equality by erasing race from public discourse. Without the means to explicitly describe and measure the experiences of racialized people, the structural effects of racism remain unacknowledged and unaddressed, further entrenching racial marginalization [30].

Importantly, while race is a socio-political construct and its misuse has historically perpetuated harmful stereotypes, it is still a necessary variable in health research. Tracking outcomes by race allows researchers and policymakers to identify and address the very health disparities that stem from social inequalities and systemic racism.

### Defining racism: German words for race or racism

Before one can have a full understanding of the harm caused by racially biased terminology, it is necessary to define racism. Germany's Grundgesetz (Basic Law) explicitly prohibits discrimination based on race in Article 3(3), reflecting the legal acknowledgment of racism's existence and its rejection in the post-World War II era. However, the term “Rasse” as used in the *Grundgesetz* carries problematic *biological* connotations. In fact, in both legal and everyday contexts, the word “race” is still overwhelmingly understood in biological terms. This misunderstanding makes it difficult for many Germans to talk about racism without also feeling they must reject the very concept of race altogether. Although scientific evidence has long disproven the existence of biological races, and there is a well-established body of German scholarship on racism, particularly from Black and Afro-diasporic perspectives [46], much of this research has failed to gain traction, especially within medical and clinical discourse [74]. This gap in uptake has contributed to persistent conceptual confusion and a lack of shared vocabulary in healthcare settings, which in turn limits the ability of providers to recognize and address systemic discrimination.

While the term "Rassismus" also exists in German, it is often poorly defined or misunderstood, particularly in medical discourse. Although structural and institutional forms of racism have been extensively theorized in German scholarship (e.g., [74]), these insights have not been widely adopted in clinical contexts. There is the same reluctance and lack of shared vocabulary to describe its structural, systemic and institutional forms. This lack of conceptual clarity has hindered the development of effective anti-racism efforts in clinical settings, where racism is often reframed as cultural misunderstanding or communication breakdown.

Here we define “racism” to mean a system that results in benefits to the in-group at the expense of the out-group, based on the notion of race. Racism is a system of beliefs (racial prejudices), practices (racial discrimination), and policies that operate to advantage those with historical power, which is White people in most Western nations [33].

As such, the term "Morbus Mediterraneus" reflects a racially prejudiced viewpoint that patients from these regions are inherently more dramatic or expressive about their pain, and also that their complaints are less legitimate than those of White Germans. This in turn, we argue, leads to a deficit in pain management and care that disproportionately affects non-White people in Germany, who may or may not be from the Mediterranean.

Racial discrimination is a pervasive issue in Germany, affecting various aspects of life, including employment, education, and healthcare [7]. According to a 2022 survey by DeZIM e.V. [70], the vast majority of Germans (90%) acknowledge the existence of racism, with nearly half viewing it as a force that shapes daily life and social institutions. More than 80% of respondents recognized discrimination in sectors such as education, employment, and housing. However, in a contradictory sentiment, the survey also found that over one-third of participants (33%) viewed those affected by racism as overly sensitive, while more than half (52%) felt that anti racism efforts unduly limit freedom of speech and may be excessive. Notably, defensiveness about racism was highest among those aged 55–64, while the youngest group (14–24 years) showed the least defensive attitudes [64].

In other words, the overwhelming majority of Germans recognize that racism exists and impacts the lives of those who are racialized, but at the same time, between a third and a half of the same people, downplay, dismiss or trivialize the issue. This shows that the issue is obvious and visible on its face, while the consequences and repercussions of racism are nonetheless minimized.

### Terminology gap impedes scholarship on racism

The historical confusion around the meaning of terms describing race and racism in German scholarship has created an impediment to their study. Gerhards and colleagues [29] explain how a limited understanding of the functions and types of racism among German students hampers their ability to recognize, reflect on, heal from, or address it. Medical students in their research study experienced and even perpetuated forms of racism but struggled to name or categorize these behaviors, highlighting the consequences to this critical gap in Germany's academic discourse.

The absence of appropriate language and measurement categories prevents researchers and policymakers from fully identifying or quantifying these impacts. In health research, race and ethnicity are frequently used as classification variables, though often inconsistently and inaccurately. However, race has become a necessary variable in medical research because it has an impact on life trajectory, opportunities, and health outcomes [40]. Therefore, the ability to classify groups of people based on race and measure outcomes can reveal health disparities stemming from social inequalities. Although race data is standardly tracked in the U.S., the European Union and Canada have only recently begun encouraging the use of race and ethnicity data to better quantify and combat healthcare inequities [51].

### Historical underpinnings

The idea that people of color feel less pain is not new to the German medical establishment. In fact, Nobel Laureate Heinrich Hermann Robert Koch, renowned for his foundational work in microbiology, conducted unethical research in what was then German East Africa and Togo during the era of German colonialism around the turn of the century [84]. While investigating diseases such as sleeping sickness, he placed thousands of Indigenous Africans into concentration camps and conducted dangerous experimental treatments on them by force, reflecting the pervasive racial biases of the time [11, 24]. These experiments included the administration of arsenic, which resulted in pain, disfigurement, blindness, and death [24, 67]. Such practices were underpinned by misconceptions that African people experienced pain less intensely than White Europeans, justifying a disregard for their suffering and well-being. Although it is clear today that this research was inhumane and a violation of ethical principles, the beliefs and assumptions that facilitated this work have been carried forward into the twenty-first century, and, as we will discuss, continue to exist in the unconscious if not conscious minds of practitioners today [38]. Koch’s scientific accomplishments are highly celebrated in Germany and across the world. The medical establishment today is too willing to forget his crimes, and the atrocities he committed in East Africa are rarely mentioned [11]. He not only harmed thousands of people, but his efforts contributed to the widespread acceptance of the idea that in medicine, different rules apply to Black and White human beings.

These beliefs were not unique to Germany. Across colonial empires, medical institutions rationalized experimental or abusive treatment of racialized peoples by promoting the idea that they were biologically different, more resilient to pain, or less worthy of ethical consideration.

One infamous example is James Marion Sims, often called the "father of modern gynecology," who conducted surgeries on enslaved Black women in the United States without anesthesia [78]. His work, as with Koch’s, was celebrated despite being built on racist assumptions and disregard for the humanity of his subjects. These historical cases across national contexts demonstrate a transnational logic of racialized medicine that continues to shape implicit biases and treatment disparities today.

### Racial disparities in pain management

Although German data on race and pain management are limited, research from the United States reveals persistent and well-documented disparities. A study presented from John Hopkins at the 2024 American Society of Anesthesiologists’ annual meeting identified significant racial disparities in post-surgery pain management, with Black patients less likely to receive multimodal analgesia compared to White patients [54]. Multimodal analgesia, an approach that combines various pain medications, is associated with better pain control and reduced opioid dependency [26]. However, Black patients in the study were 74% more likely than their White counterparts to be prescribed opioids alone, reflecting a more limited and potentially riskier treatment plan.

Similar disparities exist in obstetric care. Hispanic and non-Hispanic Black patients are more likely to receive general rather than regional anesthesia during Cesarean deliveries and are less likely to have their postpartum pain adequately assessed or treated with opioid analgesia [49]. These disparities are not due to biological differences but are shaped by systemic biases in pain management protocols [58, 77].

Some medical professionals simply have inaccurate beliefs about BIPOC physiology due to historical racist notions going back to Robert Koch, the legacy of global colonial medicine, ongoing lack of education, and poor training [38, 73]. These beliefs falsely suggest biological differences, despite the lack of scientific evidence. Even more concerning are clinical practices that adjust diagnostic thresholds based on race, practices now widely discredited. Such misconceptions include the ideas that Black people have thicker skin, need higher radiation doses during x-ray exposures due to supposedly denser bones, or have racially influenced organ functions. For example, kidney function was historically estimated with a race culturaladjustment in eGFR equations, based on the assumption that Black individuals have higher muscle mass and thus higher baseline creatinine levels. These assumptions led to race-based adjustments in lung-function and kidney-function equations, which systematically downplayed disease severity in Black patients and delayed access to care. These practices have been widely criticized and are now being removed from major clinical guidelines in favor of race-neutral algorithms [8, 9, 23].

These misconceptions are not just theoretical—they result in real harm. Healthcare providers may unconsciously assume that BIPOC patients can tolerate more pain due to cultural stereotypes or assumptions about their life experiences [6]. These biased beliefs are sometimes framed as compliments (e.g., “*your people are so resilient*”) [43], yet they lead to the systemic undertreatment of pain. Because these assumptions often operate below the level of conscious awareness, simply instructing providers to treat all patients equally is insufficient. Addressing these disparities requires acknowledging the historical roots of these biases and confronting the cultural narratives that sustain them.

Table 2 provides two lived examples, one from a patient, one from a trainee, that illustrate how these biased assumptions manifest in clinical care. These vignettes underscore how racialized beliefs about pain, even when not explicitly voiced, can result in diagnostic dismissal, substandard care, and moral injury to patients and providers alike.

**Table 2:** Lived experience: patient and provider anecdotes

Patient Experience	Provider Experiences
Native German Representative Mirrianne Mahn, a representative (Stadtverordnete) from the city of Frankfurt. She tearfully recorded herself from her hospital bed in December 2020 after being admitted for acute pain to a Frankfurt German Hospital [53]. She recounted in the video that the doctor, who continually referred to her as “the African” rather than by her name, said, “*Be glad you are here, because with your ailments you would be dead by now if you were in Africa* […] then I asked him if the pain was normal, and he said *pain is never normal, but your compatriots [you Black people] can put up with it better; if you were in Africa, it would be much worse*." She went on to say, “It's terrible to be put at the mercy of these people, they don't see you as a human being … in any other situation, I wouldn't put up with it, but simply when you're in hospital you're dependent on them…” Compounding the injury caused by the explicit racist behavior of the physician, her video posted publicly garnered hundreds of views but also a barrage of hateful racist comments. Bowing to public pressure, the doctor later apologized for his remarks	In 2017, an anesthesiologist at a hospital in Hamburg was paged to place an epidural for a woman of Turkish heritage in active labor. Nurses had repeatedly documented her complaints of severe pain but expressed skepticism, stating she was “likely another Morbus Mediterraneus case.” When they briefed the anesthesiologist, they referred to her as “dramatic” and implied her pain was exaggerated. However, upon examination, he noted that her vitals and visible distress were consistent with significant pain. Recognizing the validity of her complaints, he quickly administered an epidural and ensured adequate monitoring. In retrospect, the physician documented the use of the term “Morbus Mediterraneus,” concerned that casual racism had nearly led to underestimating the patient’s legitimate need for relief
	“Ben” (name changed for anonymity), a medical student from Berlin, observed an incident where a woman with limited German language skills was presented with abdominal pain in the emergency room. Unable to specify the exact location or intensity due to language barriers, she was discharged with barely any diagnostic testing and only a mild painkiller. The attending doctor referred to her case as "*Morbus Mediterraneus*" to Ben, revealing a dismissive attitude rooted in prejudice. Ben observed that such terms were routinely used by both medical and nursing staff, often behind the patients' backs. He felt that patients perceived as "German" (White) received more thorough examinations

This table illustrates how racial and gender biases in pain perception manifest in clinical practice, drawing from firsthand accounts by a patient and a medical trainee. These lived experiences highlight the real-world consequences of provider bias, including diagnostic dismissal and unequal treatment. The anecdotes reflect long-standing racialized and gendered stereotypes—unsupported by scientific evidence—that continue to shape care despite having no basis in human biology

This problem also emerges in medical education. A study by Gerhards, Schweda, and Weßel [29] examines German medical students' perspectives on racism within medicine and healthcare, identifying critical gaps in understanding and addressing racial issues in medical education. Through interviews and discussions, the researchers highlight that while many students recognize racial discrimination in clinical settings, there was uncertainty on how to address them, and some students struggled to balance medical classification practices with the risk of reinforcing racial bias. One particularly telling response captures this tension. Some students labeled the use of the term "Morbus Mediterraneus" as “super racist,” while others defended it as “very practical,” justifying racial and cultural classifications as a necessary part of medical training. As one student put it regarding diagnosing patients by race, “there’s no other way to do it.” reflecting a concerning normalization of racialized clinical thinking [29].

However, this approach only embeds bias into patient care by justifying racialized terminology under the guise of practicality, ultimately reinforcing false stereotypes. The same study called for enhanced medical education to better equip students to recognize and resist the use of racialized classifications in practice. Without this, future clinicians risk reinforcing the very disparities they are being trained to resolve.

### Implications of the term

The term “Morbus Mediterraneus”, also called “Morbus Bosporus” or “Balkan Syndrome”, is a pseudoscientific ailment that is actually a designation used by medical professionals shaped by racist and stigmatizing prejudices. Lacking any scientific basis, it describes an invented phenomenon whereby patients, typically women from Southern regions, are assumed to have a lower pain threshold than White Europeans and are generally more sniveling and overdramatic in their complaints [82].

This reflects a clear case of intersectionality, where race and gender interact to shape the way pain is perceived and treated. As Crenshaw [19] explains, intersectionality describes how multiple systems of oppression, such as racism and sexism, intersect to produce unique and compounded disadvantages. In the case of "Morbus Mediterraneus," racialized women are doubly stigmatized: their pain is dismissed as exaggerated not only because of cultural stereotypes, but also due to gendered assumptions that women are overly emotional or unreliable narrators of their own suffering. This dual bias increases the risk of misdiagnosis, inadequate treatment, and psychological harm [12].

This can lead to measurable discrimination against people of color by medical staff, for example through less attention to symptoms, hasty diagnoses, disrespect in communication, or even being sent away prematurely. Further, even within the terminology of the diagnosis, the “Mediterranean” portion of the word is not a clearly delineated geographic region. Rather, in practice the term refers to people who are considered non-White.

By relying on vague geography to signal race and cultural difference, the term obscures its own function as a racial marker. As such, people of color experience being reduced to their cultural or ethnic origin and are no longer perceived as individuals but as representatives of a homogeneous and inferior group.

The paradox is that, as explained above, there is a historic racist idea that BIPOC can withstand superhumanly more pain than White people [38, 39, 79], and therefore are treated with insufficient analgesics, and made to suffer. Yet at the same time, BIPOC are considered cowardly because they claim they cannot bear the pain that a White person supposedly could and are therefore punished by having analgesics withheld [60]. In other words, racialized patients are placed in a double bind: they are either pathologized for expressing pain or disbelieved when they do not.

There is no scientific justification for either of these notions, yet medical decisions are being made simply based on these prejudices. Only suffering seems to be the common denominator and Whiteness the “golden mean” – midway between super tolerance and over sensitivity, where Whiteness is the only real reason a patient deserves to be properly treated for pain.

The use of "Morbus Mediterraneus" signifies a broader issue of racial and gender biases in healthcare. In clinical practice, it undermines the trust between patients and healthcare providers and can lead to serious health consequences due to inadequate assessment and treatment. It also perpetuates a climate of casual dehumanization, where discriminatory terms are normalized in clinical routines. By homogenizing and devaluing a group of patients based on ethnicity and gender, the term exemplifies systemic discrimination within the medical field.

## Cultural differences in pain expression

### Influence of cultural background

Cultural norms profoundly shape how individuals perceive, express, cope with and respond to pain [20, 41, 72, 83]. In collectivist cultures, such as those prevalent in Mediterranean and Middle Eastern societies, open expression of pain may be more socially acceptable [18, 35]. Expressing discomfort is not merely a personal act, it can serve as a social cue to elicit care, solidarity, and community support. This is a culturally coherent and adaptive practice that should not be misread as dramatization or hypersensitivity [35, 48]. Further, being honest about one’s experience of pain is an important means of eliciting the support needed from the community to recuperate.

However, in medical contexts shaped by Western norms of stoicism and restraint, such expressions may be misinterpreted [48]. Healthcare providers, especially those unfamiliar with the patient's cultural background, may view emotionally expressive behavior as exaggerated, manipulative, or lacking credibility. This misreading is not benign. It can result in inadequate diagnostic efforts, delays in treatment, and a reduction in pain management interventions, thereby contributing to avoidable suffering [72].

Cross-cultural research consistently shows that cultural identity influences not only how pain is expressed but also how it is perceived by observers [36]. German cultural frameworks, in particular, tend to emphasize self-discipline and stoicism as honorable traits, which may discourage open displays of pain [88]. In contrast, patients from cultures with strong familial and communal values often express pain openly to communicate distress and activate support networks—behaviors aligned with cultural norms, not signs of pathology.

As introduced earlier, [20] found that Hispanic/Latine individuals reported higher pain severity than their White counterparts, consistent with U.S. trends. However, cultural factors such as religious beliefs, internalized stoicism, and fear of opioid dependence shaped how participants interpreted and reported their pain. These factors often led to underreporting or hesitation to seek treatment—misunderstood by providers as avoidance or exaggeration.

Misinterpretation of these expressions by healthcare providers unfamiliar with the cultural context can lead to inadequate pain management and patient dissatisfaction [72]. Indeed, they are wrongly interpreted by German medical professionals as unsophisticated attempts at getting undeserved attention in the absence of any legitimate medical needs.

Interestingly, the stereotype of lower pain tolerance among non-White patients is not upheld by empirical findings. A study of ethnically Turkish and German birthing mothers showed that Turkish women were less likely to request epidural analgesia than White German women [69]. Notably, the authors concluded that the Turkish women refused epidurals due to “misconceptions” about potential complications but failed to consider the role of cultural values, patient agency, and prior negative interactions with healthcare systems. For many racialized patients, distrust in medical institutions is not irrational, but a learned response to repeated marginalization.

Language barriers further complicate communication about pain [36]. Patients with limited proficiency in the dominant language may struggle to describe their symptoms clearly or in medically acceptable terms [50]. This can lead to clinician frustration and the dismissal of complaints as vague, exaggerated, or psychological in origin [50]. Instead of viewing these situations as evidence of patient unreliability, providers must learn to recognize them as moments that call for empathy, interpretation, and culturally competent care. Additionally, research has shown that providers may unconsciously associate foreign accents or limited language proficiency with lower intelligence, compliance, or credibility, which further entrenches negative stereotypes [50].

Some patients also report that clinicians avoid switching to English or simplifying language even when asked, which reinforces the power imbalance and leaves patients feeling disrespected or ignored Patients of color have reported that racism makes some professionals unwilling to speak in English to meet their needs if they are not fluent in German [22]. These dynamics can deepen mistrust, exacerbate disparities, and contribute to long-term disengagement from care.

As noted earlier, our recent study [20] found that racialized individuals, particularly Hispanic/Latine Americans, reported greater pain severity than non-Hispanic White participants. This relationship was partially mediated by lifetime experiences of racial discrimination, underscoring how the cumulative stress of racism contributes to disparities in pain. Individuals who used avoidant coping strategies in response to discrimination reported especially high pain levels, highlighting the need for trauma-informed and culturally responsive care. These findings further support the conclusion that expressions of pain among people of color are rooted in real, complex, and racialized lived experiences—not exaggeration.

Clinician-patient cultural mismatch can also exacerbate these disparities [20, 36, 48, 72]. Providers lacking cultural knowledge may misjudge pain severity or fail to interpret pain-related communication accurately, further worsening outcomes for racialized patients. This underscores the importance of not only interpreter access but also culturally matched or trained providers in pain management contexts.

### The problem of German cultural stoicism

German cultural stoicism refers to a perceived or real cultural trait rooted in historical and philosophical influences within German society. Such traits should be understood as socialized and develop independent of race [16, 45]. Stoicism, originally an ancient Greek philosophy, emphasizes self-control, rationality, and endurance in the face of adversity. It suggests a collective attitude where emotional restraint, personal resilience, and a focus on duty and practicality are valued. People may be praised or admired for enduring great suffering. In the context of German culture, this form of stoicism can manifest as a tendency to approach life with discipline, a strong work ethic, and an emphasis on maintaining composure during hardship, qualities often praised as mature or respectable. These values can shape both how pain is expressed and how it is interpreted, especially in clinical contexts.

While we refer to “German cultural stoicism,” we do so with the understanding that German society is not culturally homogenous. These observations reflect dominant norms observed in institutional or clinical settings and do not capture the full range of perspectives or lived experiences present in Germany’s diverse population. Highly valuing emotional restraint and self-control in the face of distress is a form of stoicism found in German culture, which while not universal, reflects a shared understanding of what constitutes respectable behavior, especially in public or institutional settings. This orientation begins in early childhood. A cross-cultural study comparing German and Turkish preschool children found that even at ages 3 to 6, German children are socialized differently, demonstrating more autonomy-oriented self-concepts, shaped by cultural expectations around individual responsibility and independent coping [16]. Recent cross-cultural research suggests that many Germans associate honor with fulfilling professional duties, adhering to social norms, and maintaining composure in the face of adversity [4, 45]. In medical contexts, these expectations may subtly influence how patients express pain and how clinicians interpret it.

In a comparative study of honor and dignity in India and Germany, German participants linked moral integrity and the fulfillment of duty to the preservation of social reputation yet expressed discomfort with overt emotional displays or discussions of personal hardship [45]. Unlike Indian participants, who described honor in more relational and expressive terms, German participants tended to view stoicism as a personal virtue, especially among men, and considered it more honorable to endure suffering quietly than to seek attention or communal support. Further in German professional groups, especially among physicians, honor and respect have been tied to ethical self-regulation and composure, reinforcing the social value placed on restraint [4].

Rather than encouraging open expressions of discomfort, these cultural expectations may reinforce the underreporting of suffering—a pattern sometimes misread as emotional resilience or maturity. In professional settings, particularly among healthcare workers, stoic demeanor is often associated with ethical self-regulation and social responsibility [45]. While this norm may promote discipline and professionalism, it can also obscure distress and lead to disparities in care for individuals whose cultural norms permit more expressive communication.

The idea that pain makes one stronger, though culturally resonant in Germany, is not a scientific truth. While moderate physical challenges can foster resilience, chronic or unaddressed pain can lead to serious health consequences. Patients may internalize cultural expectations of endurance, underreporting or delaying disclosure of symptoms in order to appear strong or self-reliant. This can lead to missed diagnoses and undertreatment of conditions such as cancer, where pain may only be acknowledged at an advanced stage.

Moreover, clinicians shaped by the same cultural values may unintentionally minimize patients' pain or view emotional expression as immature or destabilizing. A restrained demeanor may be interpreted as trustworthy, while more emotive presentations may be perceived as exaggerated or unprofessional. This interpretive bias becomes especially problematic in cross-cultural settings.

When such patients are racialized as non-German, particularly those of Southern European, Middle Eastern, or North African descent, clinicians may hastily label them with “*Morbus Mediterraneus*,” a stigmatizing term that reflects both cultural and racial bias. This label not only delegitimizes the patient’s experience of pain but also frames emotional expression as pathological or culturally inappropriate. Culturally competent care must recognize that neither stoicism nor expressiveness is inherently superior, and that medical objectivity requires attention to the full spectrum of human pain expression.

Though widely accepted in Germany, the idea that physical pain makes you stronger is primarily a cultural belief or motivational adage. While moderate physical challenges can lead to increased resilience and adaptation, severe or chronic pain does not make one stronger and can have significant negative consequences.

While cultural stoicism can promote admirable traits like resilience and patience, it also contributes to a culture where pain is undertreated or misunderstood. Patients may underreport or downplay their pain based on a desire to embody the values of endurance and resilience. They might view expressing pain as a sign of weakness or a failure to maintain composure, which can lead to delays in seeking medical help or receiving adequate treatment due to pain (e.g., pain from cancer may not be detected until in a late stage due to this approach).

Cultural norms that stigmatize the expression of pain can lead to significant negative patient health outcomes. Those conditioned from an early age to suppress or ignore bodily discomfort may develop a heightened tolerance for pain or feel compelled to endure symptoms without complaint. This can lead healthcare providers to misinterpret a patient's calm demeanor as an indication of minimal distress, potentially resulting in misdiagnosis or underestimation of pain levels. In *The Myth of Normal,* Gabor Maté [55] warns that disconnection from the body's signals hinders recognition of health issues delaying care and increasing the risk of chronic illness. These insights underscore the need for culturally informed communication in clinical settings.

Medical professionals influenced by the same cultural norms might also under-prioritize pain management. They may unconsciously adopt the belief that enduring pain is natural or expected, potentially leading to less aggressive pain relief strategies and a preference for practical over compassionate approaches. Moreover, Patients who express pain emotionally with crying, moaning, or grimacing may be dismissed as weak, childlike, or emotionally immature. When such patients are non-White, providers may be too quick to label the problem as “Morbus Mediterraneus.” which compounds bias with stigma.

## Implicit bias and racism in healthcare

### Implicit racial bias

Implicit biases are unconscious attitudes or stereotypes that affect understanding, actions, and decisions without conscious awareness. In healthcare, these biases can influence clinical judgments in subtle but significant ways, leading to disparities in treatment, diagnosis, and communication. One of the most heavily studied forms of implicit bias is implicit racial bias, particularly anti-Black bias [7]. These biases are pervasive [31], as has been demonstrated with global studies using the Implicit Association Test (IAT [62]), for racial bias. Healthcare providers are not immune to implicit bias, and in fact often harbor equivalent or more racial biases when compared to the general population [85]. These biases have been shown to negatively impact the health outcomes of people of color as well as influence how they are treated by White providers [31, 52, 66].

Maina and colleagues [52] conducted a systematic review examining provider bias in healthcare. Six of seven studies found that higher implicit bias was associated with racial disparities in treatment recommendations, expectations of therapeutic bonds, pain management, and empathy, and all seven found that providers with stronger implicit bias demonstrated poorer patient-provider communication. While most of these studies used cross-sectional designs, limiting the ability to infer causality, all seven studies were unanimous in finding that providers with stronger implicit bias exhibited poorer patient-provider communication with their patients of color.

### The empathy gap

Implicit biases can lead healthcare providers to underestimate the pain of patients of color or assume that they are exaggerating their symptoms, resulting in undertreatment. As noted, research has shown that people have less empathy for those outside their own racial group, such that the physical pain of people with a different skin color is underestimated, and this effect is higher in people with more implicit bias [6, 10].

**Fig. 1:**
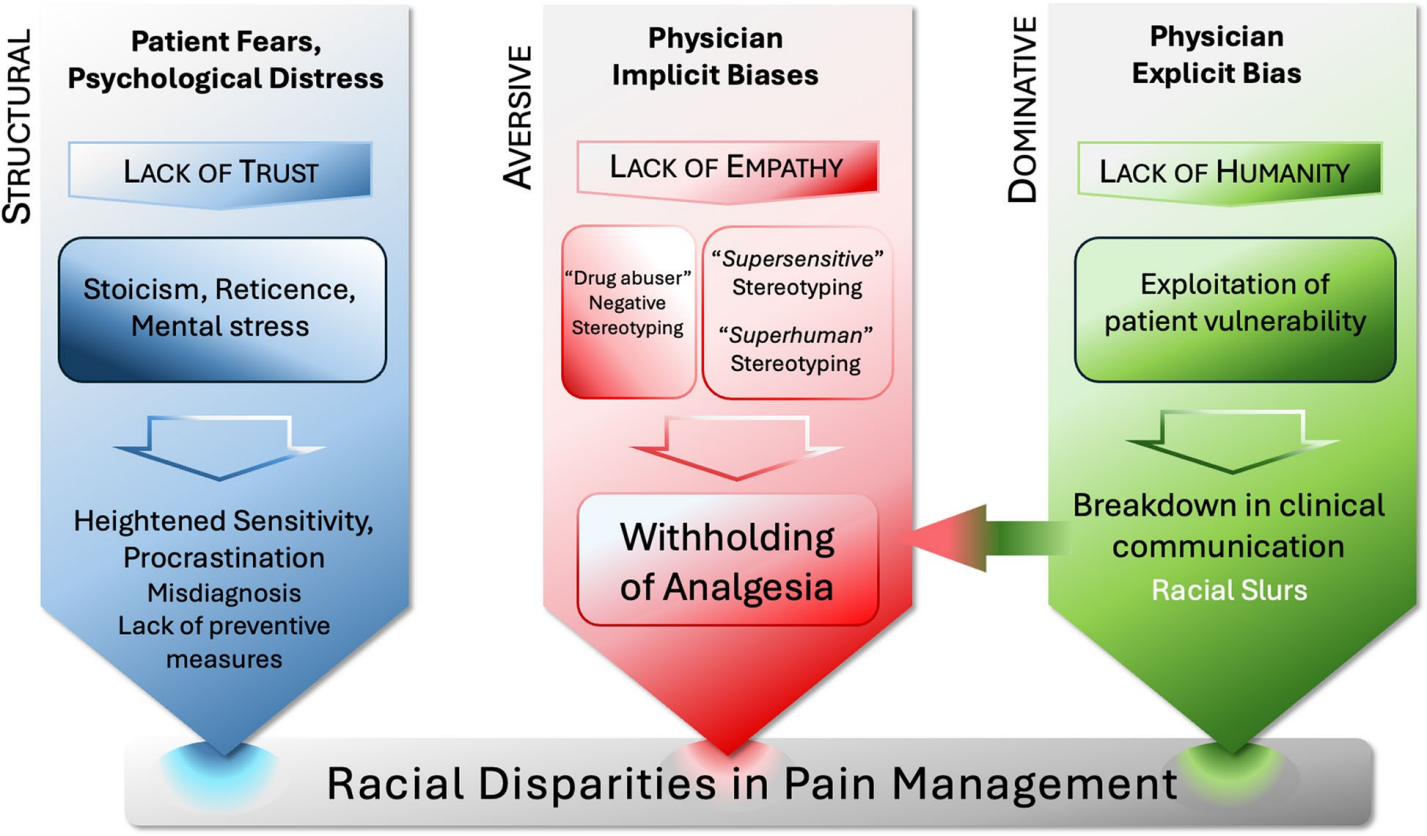
Racial disparities in pain management. Note: Core Issues leading to disparate outcomes in pain management. Three different types of racism, structural, aversive, and dominative, lead to different types of downstream issues, but all contribute to racial healthcare disparities in pain management

Recent studies in Germany have produced similar findings. For example, Fabi and Leuthold [27] found that White German students showed stronger empathic brain responses when viewing images of pale-skinned hands in pain compared to dark-skinned hands. These differences were correlated with implicit bias scores on the Implicit Association Test, suggesting that racial bias influenced both neural and cognitive empathy responses. Such biases in clinical settings can contribute to widespread disparities in pain treatment. Negative racial stereotypes, such as assumptions that patients of color are exaggerating or drug-seeking, can result in inadequate pain relief, the withholding of analgesia and increased suffering [56, 64, 71] (Fig. 1).

### Patient-provider relationships

Biases adversely affect the trust and communication essential for effective healthcare. Discriminatory treatment often comes in the form of dismissive or disrespectful treatment from providers and administrators and may include being talked down to under the assumption that such patients are poorly educated, not fluent in German, or are ignorant. These negative encounters create a dislike of providers, and as such patients will fail to return for follow-up appointments and not adhere to treatment recommendations, compared to White patients [59, 68]. It can also result in negative perceptions of the healthcare system overall, prompting racialized patients to delay seeking medical attention for future problems. This delay can result in the worsening of pain conditions as early intervention is critical for effective treatment and management and ultimately creates a health disparity along racial lines. Figure 1, adapted from Penner et al. [68], illustrates this process.

In sum, racialized patients receive less adequate pain management compared to their White counterparts. Anderson et al. [86] found that such disparities are caused by a combination of patient-related factors, provider biases, and systemic issues within healthcare institutions.

## Psychological impact of discrimination on pain perception

### Pain sensitivity and chronic pain conditions

As described in the previous sections, such disparities are rooted not in individual failings but in the legacies of colonial and structural racism embedded in medical institutions. The cumulative stress of racism on the physical health and emotional well-being of racialized persons is well-documented [63, 75]. Stress can heighten pain sensitivity and its severity. As such, the stress associated with experiencing race-based discrimination (Fig. 1) can also significantly contribute to the experience of pain [37]. This finding is supported by U.S. studies showing that racialized groups, particularly Black and Asian Americans, who report greater pain intensity and interference [28, 47]. Similar trends have been observed in Germany among individuals categorized as having a ‘migrant background’ (e.g., [44]).

This increased pain sensitivity is not biological, but rather a biopsychosocial consequence of chronic exposure to racism and marginalization. Studies suggest that depression and other forms of psychological distress partially mediate this relationship, leading to phenomena such as central sensitization [76]. Among Black Americans, for example, lifetime discriminatory experiences have been shown to be a stronger predictor of chronic back pain than any other variable, especially in women (25).

Racialized patients may not only experience more severe pain but also require more aggressive treatment due to the cumulative toll of discrimination which is an effect resulting from structural inequity and lived social realities, not biological differences [17, 37].

### Mental health considerations

Physical pain can significantly contribute to poor mental health due to the close link between pain and emotional well-being. Persistent pain can lead to stress, frustration, and feelings of helplessness, which, over time, may develop into mental health conditions such as major depressive disorder and anxiety. The continuous strain of dealing with pain can disrupt sleep, limit daily activities, and reduce overall quality of life, creating a cycle where physical pain worsens emotional distress and vice versa [5]. Additionally, the brain processes involved in pain and emotional responses often overlap, meaning that ongoing pain can directly impact the brain's ability to regulate mood and stress, further exacerbating mental health problems [57, 65].

As such, addressing both psychological and physical aspects of pain is essential. The intersection of race, pain, and mental health requires holistic treatment approaches that consider the patient's experience and cultural context.

## Addressing "Morbus Mediterraneus" in medical education and practice

### Need for cultural competence training

In the medical profession, provider lack of empathy is life-threatening, yet a lack of empathy continues to drive substandard care for people of color in Germany. Many well-meaning individuals and organizations feel helpless to bring about change as the behaviors driving these problems are covert and typically denied.

Ben's experience illustrates typical biases and deficiencies in training, yet this critical learning is needed to provide racism-free care. The good news is that research shows that effective training can be implemented to improve provider behavior and patient health outcomes. Mandatory seminars and workshops can equip future and current healthcare professionals with the skills to provide equitable care.

For example, Kanter and colleagues [42] tested the Racial Harmony Workshop (RHW; [81]) with medical students to ascertain if the intervention could improve the interracial patient-provider relationship when racial issues were salient. This randomized trial employed a therapeutic model from social and contextual behavioral sciences based on functional analytic psychotherapy (FAP), a relational approach that emphasizes empathy and openness. The intervention specifically aimed to decrease providers’ likelihood of expressing biases and negative stereotypes when interacting with patients of color in racially charged moments, such as when patients discuss past incidents of discrimination. Workshop exercises were informed by research on the importance of mindfulness and interracial contact involving reciprocal exchanges of vulnerability and responsiveness. The study documented improvements in observed emotional rapport and responsiveness, fewer microaggressions, decreased racism toward minoritized groups, and improved self-reported working alliance and closeness with the Black standardized patients in the study. This suggests that interventions that directly intervene to help providers improve responding in these moments can be beneficial in reducing racial health disparities.

There are several organizations in Germany that can provide clinician anti-racism training in the workplace, including the Phoenix e.V., AWO Bundesverband, and the Amadeu Antonio Stiftung.

### Beyond cultural competence: embracing structural competence

While cultural competence training remains valuable, it has been critiqued for sometimes essentializing cultural differences and failing to address the broader social, political, and economic structures that shape health outcomes. As Metzl and Hansen [87] argue, what is often needed is structural competence—the trained ability to recognize how systemic factors such as institutional racism, economic inequality, and policy decisions produce health disparities. Integrating structural competence into medical education encourages providers to move beyond individualized understandings of cultural difference and instead develop tools to analyze and address the upstream causes of health inequities. Including both cultural and structural perspectives in training ensures that interventions do not simply focus on interpersonal bias but also equip providers to recognize and challenge the institutional patterns that perpetuate harm.

### Reducing implicit biases

Awareness of implicit biases can lead to more conscious, equitable decision-making in pain treatment [59]. To reduce implicit biases, healthcare providers must engage in their own personal self-reflection and bias mitigation strategies (e.g., [15]) and consider ways they can ensure they are treating everyone fairly. Implementing standardized pain assessment tools is one practical and simple way to reduce subjective judgments influenced by biases.

### Enhancing diversity in healthcare

Increasing diversity among medical professionals can improve patient-provider communication and trust, as clinician-patient racial concordance positively influences health outcomes [86]. Further, when there is a diversity of providers, they can learn from each other how to be more sensitive and informed. Facilities should take stock of the diversity of their staff and hire more racialized providers if their compliment is not representative of their region. However, this must also include reducing racism in the workplace so that racialized medical professionals can function without harm [14].

### Providers should address racist behaviors in clinical settings

When clinicians hear colleagues use terms like "Morbus Mediterraneus," this is an opportunity to educate and provide corrective information. In fact, it is a clinician’s ethical duty to address this issue to prevent harm to current and future patients. Clinicians should take proactive steps to address the issue professionally by initiating a respectful dialogue with the offender. They can approach the colleague privately and explain that such language can perpetuate stereotypes, negatively impacting patient care. Emphasize the importance of using current, evidence-based medical terminology, without relying on outdated stereotypes. Provide current information about racialized groups, including the fact that there is no scientific evidence that they exaggerate pain symptoms. Emphasize the importance of taking the pain reports of all patients seriously. Appeal to the colleagues’ higher morals and say things like, “Let’s do the test to be sure. Wouldn’t you feel awful if we missed something really important going on?”.

If this is a consistent problem, it would be a good idea to encourage the colleague to engage in anti-racism or cultural-sensitivity training. You can also provide articles and other resources on unconscious bias in healthcare (e.g., [15, 80]). Sharing educational materials can help raise awareness about the implications of discriminatory language and snap judgements about people. If the colleague's behavior continues or reflects a broader pattern of discrimination, consider reporting the issue to a supervisor, ethics committee, or the institution's diversity and inclusion office.

Ben’s experience highlights the challenges of addressing discriminatory behavior in clinical settings, especially as a trainee. He could have taken several steps to address the issue, such as seeking guidance from a mentor, documenting the incident, or advocating for institutional anti-bias training. Additionally, he might have engaged in peer education, reported the behavior through formal channels, or respectfully challenged the comment in the moment to emphasize evidence-based care and equitable treatment.

Due to power differentials, in some cases it may not be possible to confront offenders. In these cases, the clinician should make an anonymous complaint. Even if it is not the institution’s policy to act on anonymous complaints, if there are several such complaints, they will eventually be noticed. Additionally, there are supports for medical providers experiencing or witnessing racism. In addition to the federal “Antidiskriminierungsstelle” for example, “*Wir sind vielfältig/Wir sind stark*” funded by Förderverein der Arbeitsgemeinschaft der Beiräte für Migration und Integration RLP (AGARP) e.V., is a regional example of an organization that provides help to providers.

### Patients: don’t take it lying down

Patients of color who are having their pain or symptoms dismissed without proper examination are experiencing discrimination in healthcare, and as such should take proactive steps to advocate for themselves. If patients are too unwell to do this on their own, their families can help. This includes documenting all interactions with healthcare providers; requesting thorough explanations for diagnoses and treatment decisions and asking for evidence-based justifications; if unsatisfied, clients should seek second opinions from other professionals. Patients can also consult patient advocacy organizations for support and guidance on how to address potential biases. It helps if patients know their rights under laws such as the General Act on Equal Treatment (Allgemeines Gleichbehandlungsgesetz, AGG), which empowers them to file formal complaints with medical institutions or licensing boards if necessary. Importantly, the Federal Anti-Discrimination Agency [3] has clarified that the AGG applies to Behandlungsverträge (treatment contracts) in healthcare, meaning that patients who experience discrimination in medical care are explicitly protected under this law. All of this can and should be done if patients suspect bias, even if clinicians do not explicitly use terms like "Morbus Mediterraneus" in their presence.

## Recommendations for policy and practice

### Policy initiatives

The National Action Plan Against Racism (2017) by the German government acknowledges the presence of discrimination but falls short in effectively addressing racism within healthcare systems. Achievement of equity is a multifaceted endeavor, as outlined in Table 3. There is a need for more robust policies and enforcement mechanisms to combat systemic biases (e.g., [13]). Better education to develop more cultural competence in healthcare is critical considering the impact of cultural differences on pain expression [29, 72]. Another important step toward equity is to promote greater diversity in medical schools to increase the number of racialized providers.

**Table 3:** Recommendations for equitable policy and practice

Policy Initiatives	Guidelines	Establish clear comprehensive policies to identify and eliminate racist practices in healthcare settings
	Workforce Diversity	Require all medical and nursing schools to publish diversity statistics for admissions and graduation. Engage in blinded hiring process
	Training	Require anti-racism and cultural competence training to be incorporated into medical curricula and continuing education programs
	Research	Fund research studies on the impact of racism, Islamophobia, and colonialism on health outcomes to inform evidence-based interventions
	Accountability	Penalize regions/healthcare facilities that get many complaints of racism. Recognize those doing a good job
Institutional Initiatives	Communication	Encourage patient-centered communication which includes open dialogue, active listening, and validation of patients' experiences
	Individualized Care	Create culturally informed treatment plans for pain management tailored to the patient's specific needs
	Interpreters	Employ professional interpreters and cultural mediators to overcome language barriers and improve understanding
	Training	Provide regular anti-racist lectures and workshops for staff
	Patient Surveys	Collect patient satisfaction surveys to ensure that racialized patients are getting the same quality of care as White patients. Make results publicly available annually on institutional website
Community & Initiatives	Community Connection	Engage with community leaders and organizations to understand the needs, perspectives, and issues facing racialized patients
	Health Literacy	Develop educational materials and programs in different languages to enhance patients' understanding of health issues and the healthcare system
	Empowerment	Encourage patients and their families to advocate for their needs
	Support	Raise awareness of human’s rights office as mandated by German law. Inform employees about their right to file complaints to staff specifically educated in patient advocacy for racialized individuals

## Conclusion

The use of terms such as „Morbus Mediterraneus" in German medical settings reflects deep-seated cultural racism and sexism that adversely affect patient care, particularly in pain management. Cultural differences in pain expression, communication barriers, and implicit biases contribute to inadequate treatment for patients of the global majority, resulting in life-threatening consequences. Educational efforts must reinforce the understanding that racialized differences in pain treatment are rooted in bias and social inequities—not biological or genetic differences.

Addressing these issues requires systemic changes in medical education, practice, and policy. By integrating anti-racist training, addressing implicit biases, and improving diversity within the healthcare workforce, it is possible to improve health outcomes for racialized patients. A concerted effort is needed to dismantle prejudices and build a more inclusive healthcare system that provides equitable care for those of all racial, ethnic and cultural identities.

## Data Availability

No datasets were generated or analysed during the current study.
